# Sex-specific multimorbidity clusters and all-cause mortality among community-dwelling older adults: findings from the ASPREE cohort

**DOI:** 10.1093/gerona/glag245

**Published:** 2026-10-01

**Authors:** Rong Du, Swarna Vishwanath, Zimu Wu, Aung Zaw Zaw Phyo, Suzanne G Orchard, Kerry Sheets, Michael E Ernst, Joanne Ryan

**Affiliations:** School of Public Health and Preventive Medicine, Monash University, Melbourne, Victoria, Australia; School of Public Health and Preventive Medicine, Monash University, Melbourne, Victoria, Australia; School of Public Health and Preventive Medicine, Monash University, Melbourne, Victoria, Australia; School of Public Health and Preventive Medicine, Monash University, Melbourne, Victoria, Australia; School of Public Health and Preventive Medicine, Monash University, Melbourne, Victoria, Australia; Geriatric Medicine Division, Department of Medicine, Hennepin Healthcare, Minneapolis, Minnesota, United States; Department of Pharmacy Practice and Science, College of Pharmacy, The University of Iowa, Iowa City, Iowa, United States; Department of Family and Community Medicine, Carver College of Medicine, The University of Iowa, Iowa City, Iowa, United States; School of Public Health and Preventive Medicine, Monash University, Melbourne, Victoria, Australia; (Medical Sciences Section)

**Keywords:** Latent class analysis, Sex-specific differences, Risk stratification

## Abstract

**Background:**

Multimorbidity is common in older adults, but sex differences in chronic condition clustering remain unclear. This study explored multimorbidity clusters and their associations with all-cause mortality among community-dwelling adults aged 70 years and over.

**Methods:**

This was a secondary analysis of data from 16 095 Australian ASPREE participants aged ≥70 years without prior dementia or cardiovascular disease. Fifteen baseline chronic conditions were grouped using latent class analysis (LCA). Observed-to-expected (O/E) ratios characterized conditions over-represented within clusters, and Cox proportional hazards models assessed associations with all-cause mortality.

**Results:**

Among 16 095 participants (mean age 75.3 years), 88.3% had multimorbidity at baseline; 4217 deaths occurred over a median follow-up of 10.85 years. Five clusters were identified overall: hypertension and dyslipidemia (52.1%), gout and metabolic (14.4%), depressive symptoms, osteoporosis, and frailty (10.0%), anemia and kidney disease (10.2%), and hypotension, thyroid disorder, and past cancer (13.3%). Sex-stratified analyses revealed 3 clusters in males and 4 in females. The frailty, depressive symptoms and osteoporosis cluster was associated with higher mortality in both sexes (adjusted hazard ratio [aHR] 1.56 [95% confidence interval (CI) 1.40-1.73] in males; 1.68 [1.49-1.89] in females). Higher mortality was also observed for the metabolic, gout and kidney disease cluster in males (aHR 1.63 [1.47-1.81]) and the gout, anemia and kidney disease cluster in females (aHR 1.96 [1.74-2.21]).

**Conclusions:**

Distinct multimorbidity clusters were identified overall and in sex-stratified analyses. These hypothesis-generating findings suggest the potential clinical utility of multimorbidity clusters to identify individuals at increased risk of death, but further research is needed.

## Introduction

Multimorbidity, commonly defined as the coexistence of 2 or more chronic conditions, is becoming increasingly prevalent with the aging population, posing a major challenge for public health and healthcare systems.^1^^,^^2^ The prevalence of multimorbidity rises sharply with age, affecting approximately 65% of adults aged 65 years or older and 80% of those aged 85 years or older.^2^ This growing burden is associated with poorer quality of life, functional decline, psychological distress, greater healthcare use, polypharmacy, and increased mortality.^3^ However, its management remains complex because clinical guidelines and healthcare delivery models often focus on single diseases, making it difficult to address the needs of older adults with diverse combinations of coexisting chronic conditions.^3^^,^^4^ Multimorbidity is often measured using disease count, which provides an intuitive summary of overall disease burden.^5^ Beyond disease count, cluster-based approaches can clarify the nonrandom co-occurrence of chronic conditions and identify groups with distinct condition distributions.^6–8^ Clusters may be defined using predetermined clinical groupings or identified through data-driven approaches.^7^^,^^9^ Examining their associations with subsequent health outcomes can establish their prognostic relevance and provide a basis for future research evaluating potential clinical applications.^10^^,^^11^

Existing studies of multimorbidity clusters show substantial heterogeneity.^6^^,^^8^ Nevertheless, large systematic reviews have consistently identified cardiometabolic or cardiovascular-related clusters, often mixing chronic conditions such as hypertension and diabetes, with major health events like ischemic heart disease, heart failure, and stroke.^6^^,^^8^ Less is known about multimorbidity clustering in community-dwelling older adults without major clinical cardiovascular disease, despite a large proportion of the population living to older age in relatively good health.^12–14^ Characterizing multimorbidity in this relatively healthy population may provide insight into patterns of co-occurring conditions which can be different from those among individuals with established cardiovascular disease. Moreover, among the few sex-stratified analyses conducted to date, most have been conducted in populations from Europe, Asia, and North America, and their largely cross-sectional designs have limited the examination of associations between multimorbidity clusters and subsequent health outcomes.^8^ Using data from a large cohort of relatively healthy community-dwelling adults aged 70 years or older in Australia, this study aimed to characterize sex-specific multimorbidity clusters and their associations with all-cause mortality.

## Methods

### Study population

Data came from the ASPirin in Reducing Events in the Elderly (ASPREE) trial and its ongoing observational extension study (ASPREE-XT). ASPREE was a double-blind, randomized, placebo-controlled trial that examined the effects of daily 100 mg aspirin on various health outcomes in older adults.^15^^,^^16^ The present analysis included Australian ASPREE participants, who were recruited between March 2010 and December 2014 through their usual general practitioners, and were community-dwelling adults aged 70 years or older, free from prior cardiovascular disease, dementia, or major physical disability at trial entry.^17^ At baseline and annual study visits, trained staff conducted in-person interviews to collect information on participant characteristics, health status, and medical history, followed by laboratory assessments and review of medication use. Baseline covariates included age, sex, education, residence area, living status, smoking status, and alcohol consumption.

### Assessment of chronic conditions

At baseline, 22 chronic conditions and health-related indicators were available in the ASPREE cohort, based on direct physical and laboratory measurements, self-report, and/or concomitant medication use, with thresholds defined according to clinical guidelines,^17^^,^^18^ and all were considered for inclusion. Seven indicators were excluded: Parkinson’s disease and urinary incontinence because their prevalence was below 2%, and pneumonia, deep vein thrombosis, macular degeneration, gastro-oesophageal reflux disease, and osteoarthritis because they were assessed only in a subsample, resulting in more than 50% missing data. The final analysis included 15 baseline indicators: hypertension, hypotension, diabetes, dyslipidemia, obesity, thyroid disorder, obstructive pulmonary disease (OPD), kidney disease, frailty, cancer history, bowel polyp, gout, osteoporosis, anemia, and depressive symptoms. Definitions and data sources for each indicator are provided in Table S1. All indicators were assessed at enrollment and represented participants’ baseline health status.

### All-cause mortality

All-cause mortality was defined as death from any cause.^19^ Deaths were ascertained through medical records, reports from next of kin or close contacts during routine follow-up, and linkage to the Australian National Death Index. Follow-up time was calculated from recruitment to death or censoring, defined as loss to follow-up or the last study visit, whichever occurred first. For the current analysis, deaths up to the end of December 2024 were included in the latest release of the ASPREE-XT06 dataset.

### Statistical analysis

#### Latent class analysis

Latent class analysis (LCA)^20^ was used to identify multimorbidity clusters based on the co-occurrence of chronic conditions in the overall sample and then separately in males and females. LCA is a person-centered probabilistic approach that classifies individuals into mutually exclusive latent groups according to similar response clusters.^20^ In the overall sample, models with 2-10 classes were evaluated, whereas separate models with 2-8 classes were assessed in males and females. This range was selected to allow sufficient exploration of potential class solutions while avoiding overly complex or unstable models with small class sizes, particularly in the sex-stratified analyses. The optimal number of classes was selected based on model fit indices, including Akaike’s information criterion (AIC), Bayesian information criterion (BIC), log-likelihood, and the likelihood ratio chi-square statistic (χ²); classification quality, assessed using entropy, classification error, and average posterior probabilities (AvePP); class size; and clinical interpretability (Table S5).^11^

After class selection, the identified latent classes were further characterized according to the distribution of indicators within each cluster. For each indicator, its observed prevalence within a cluster was compared with its expected prevalence in the corresponding analytical sample. The observed-to-expected (O/E) ratio quantified relative over-representation, with values greater than 1 indicating that an indicator was more common within the cluster than expected based on its prevalence in the corresponding sample.

Cluster labels were assigned as descriptive summaries based primarily on the 2 or 3 conditions with the highest O/E ratios within each cluster. Conditions were first ranked by their O/E ratios within each cluster. When a condition was over-represented in more than 1 cluster, it was included in the label only for the cluster in which it had the highest O/E ratio. In the other clusters, that condition was excluded from the label and the next highest-ranked condition was considered. Cluster labels were not intended to provide an exhaustive description of all conditions within a cluster or to represent discrete clinical phenotypes.

#### Survival analysis

Kaplan–Meier survival curves were generated to describe survival according to multimorbidity clusters in the overall sample and in sex-stratified analyses, with between-cluster differences assessed using the log-rank test. Mortality rates were calculated as the number of deaths divided by total person-years of follow-up and expressed per 1000 person-years with 95% confidence intervals (CIs). Cox proportional hazards models were used to estimate the associations between membership in multimorbidity clusters at baseline and all-cause mortality in the corresponding analytical samples. The proportional hazards assumption was assessed using Schoenfeld residuals, and the main exposure variable satisfied the assumption. Results are presented as hazard ratios (HRs) with 95% CIs. For each analysis, the reference cluster was selected based on 2 criteria: the lowest relative over-representation of chronic conditions in the corresponding analytical sample, assessed using the O/E ratio distribution and clinical interpretability, and the lowest observed mortality incidence. Three models were fitted. Model 1 was adjusted for age and sex in the overall analysis and for age in the sex-specific analyses. Model 2 was additionally adjusted for education, residence area, and living status. Model 3 was further adjusted for smoking status and alcohol consumption.

#### Sensitivity analyses

Sensitivity analyses were conducted to assess the robustness of the cluster–mortality associations. First, the individual-level number of chronic conditions was added to the fully adjusted Cox proportional hazards models to determine whether the associations reflected multimorbidity clusters rather than simply overall disease burden. Second, to account for possible changes in disease clusters over time, we examined the association between baseline cluster membership and 5-year all-cause mortality using the same fully adjusted Cox models as in the main analysis.

Baseline characteristics and chronic conditions were summarized as *n* (%) for categorical variables and mean (*SD*) for continuous variables. Between-group differences were assessed using chi-square tests or analysis of variance, as appropriate. Statistical analyses were conducted using R version 4.4.2 (R Foundation for Statistical Computing, Vienna, Austria), with the poLCA and survival packages. All *p*-values were 2-sided, and statistical significance was defined as *p* < .05.

## Results

### Participant characteristics and prevalence of chronic conditions

Among the 16 703 Australian ASPREE participants, 608 were excluded due to missing data on at least one of the 15 chronic conditions, leaving 16 095 participants in the final analytical sample. Of the included participants, 8885 (55.2%) were female, and 7210 (44.8%) were male. Compared with males, females were slightly older, more likely to live alone, and less likely to be current drinkers or former smokers (Table 1). Overall, 14 205 participants (88.3%) had multimorbidity, defined as the presence of 2 or more of the 15 chronic conditions. Multimorbidity was more common in females than in males (90.4% vs 85.6%), and females had a higher mean number of chronic conditions (3.4 [*SD* 1.6] vs 3.1 [*SD* 1.6]).

**Table 1:** Baseline characteristics of Australian ASPREE participants in the overall sample and by sex.

Characteristics	**Overall (*N* = 16** **095)**	Male (*N* = 7210)	Female (*N* = 8885)	*p*
**Age, mean (*SD*)**	75.3 (4.4)	75.1 (4.3)	75.4 (4.4)	<.001
**Age group, *n* (%)**				<.001
70-74	9318 (57.9)	4314 (59.8)	5004 (56.3)	
75-79	4274 (26.6)	1832 (25.4)	2442 (27.5)	
≥80	2503 (15.6)	1064 (14.8)	1439 (16.2)	
**Education, *n* (%)**				<.001
≥12 years	8006 (49.7)	3818 (53.0)	4188 (47.1)	
<12 years	8089 (50.3)	3392 (47.0)	4697 (52.9)	
**Residence area, *n* (%)**				.749
Outer region	1886 (11.7)	831 (11.5)	1055 (11.9)	
Inner region	5743 (35.7)	2588 (35.9)	3155 (35.5)	
Major cities	8466 (52.6)	3791 (52.6)	4675 (52.6)	
**Living status, *n* (%)**				<.001
Living with someone	10 969 (68.2)	5761 (79.9)	5208 (58.6)	
Living alone at home	5126 (31.8)	1449 (20.1)	3677 (41.4)	
**Smoking status, *n* (%)**				<.001
Current	544 (3.4)	278 (3.9)	266 (3.0)	
Former	6582 (40.9)	3835 (53.2)	2747 (30.9)	
Never	8969 (55.7)	3097 (43.0)	5872 (66.1)	
**Alcohol consumption, *n* (%)**				<.001
Current	12 700 (78.9)	6155 (85.4)	6545 (73.7)	
Former	781 (4.9)	421 (5.8)	360 (4.1)	
Never	2614 (16.2)	634 (8.8)	1980 (22.3)	
**Chronic condition count, *n* (%)**				<.001
0	319 (2.0)	204 (2.8)	115 (1.3)	
1	1571 (9.8)	831 (11.5)	740 (8.3)	
≥2	14205 (88.3)	6175 (85.6)	8030 (90.4)	
**Chronic condition count, mean (*SD*)**	3.3 (1.6)	3.1 (1.6)	3.4 (1.6)	<.001

*p*-Values compare males and females. Continuous variables were compared using *t*-tests, and categorical variables were compared using chi-square tests.

In the overall sample and in both sexes, hypertension, dyslipidemia, and frailty were the most common conditions (Table 2), with hypertension being slightly more prevalent in males (76.4% vs 74.3% in females), while dyslipidemia (76.9% in females vs 56.8% in males) and frailty (39.7% in females vs 37.3% in males) were more prevalent in females. Among the other conditions, the most pronounced sex differences were observed for osteoporosis (9.0% in females vs 1.9% in males), gout (1.2% in females vs 7.8% in males), and depressive symptoms (11.4% in females vs 7.4% in males).

**Table 2:** Prevalence of the 15 included chronic conditions in the overall sample and by sex.

Chronic condition	Overall (*N* = 16 095)	Male (*N* = 7210)	Female (*N* = 8885)	*p*
Hypertension	12 110 (75.2)	5508 (76.4)	6602 (74.3)	.002
Hypotension	542 (3.4)	162 (2.3)	380 (4.3)	<.001
Diabetes	1587 (9.9)	871 (12.1)	716 (8.1)	<.001
Dyslipidemia	10 927 (67.9)	4095 (56.8)	6832 (76.9)	<.001
Obesity	4634 (28.8)	1869 (25.9)	2765 (31.1)	<.001
Thyroid disorder	1131 (7.0)	166 (2.3)	965 (10.9)	<.001
Obstructive pulmonary disease	1950 (12.1)	822 (11.4)	1128 (12.7)	.013
Kidney disease	3351 (20.8)	1454 (20.2)	1897 (21.4)	.069
Frailty	6215 (38.6)	2691 (37.3)	3524 (39.7)	.003
Cancer history	3133 (19.5)	1565 (21.7)	1568 (17.7)	<.001
Bowel polyp	3230 (20.1)	1574 (21.8)	1656 (18.6)	<.001
Gout	670 (4.2)	564 (7.8)	106 (1.2)	<.001
Osteoporosis	934 (5.8)	136 (1.9)	798 (9.0)	<.001
Anemia	653 (4.1)	290 (4.0)	363 (4.1)	.871
Depressive symptoms	1545 (9.6)	535 (7.4)	1010 (11.4)	<.001

Values are presented as *n* (%). *p*-Values compare males and females using chi-square tests.

### Multimorbidity clusters in the overall sample and by sex

Model fit and classification statistics for all candidate solutions are presented in Table S5. For the selected overall, male, and female solutions, entropy values were 0.411, 0.403, and 0.528, respectively, with corresponding AvePP of 0.620, 0.731, and 0.731. Five multimorbidity clusters were identified in the overall sample (Figure 1). The largest cluster (*n* = 8388, 52.1%) had the least distinctive chronic condition profile, with only hypertension and dyslipidemia being slightly more common than expected (O/E ratio just above 1). The remaining clusters showed more distinct profiles of chronic conditions. The 2nd most common cluster (*n* = 2320, 14.4%) showed high representation of diabetes, gout, and obesity, while the 3rd cluster (*n* = 1616, 10.0%) was characterized by a high O/E ratio for depressive symptoms, together with osteoporosis, frailty, thyroid disease, and OPD. Anemia and kidney disease clustered together in another cluster (*n* = 1635, 10.2%), but the 5th cluster, which had the greatest over-representation of hypotension (*n* = 2136, 13.3%), showed more moderate representation of other conditions.

**Figure 1:**
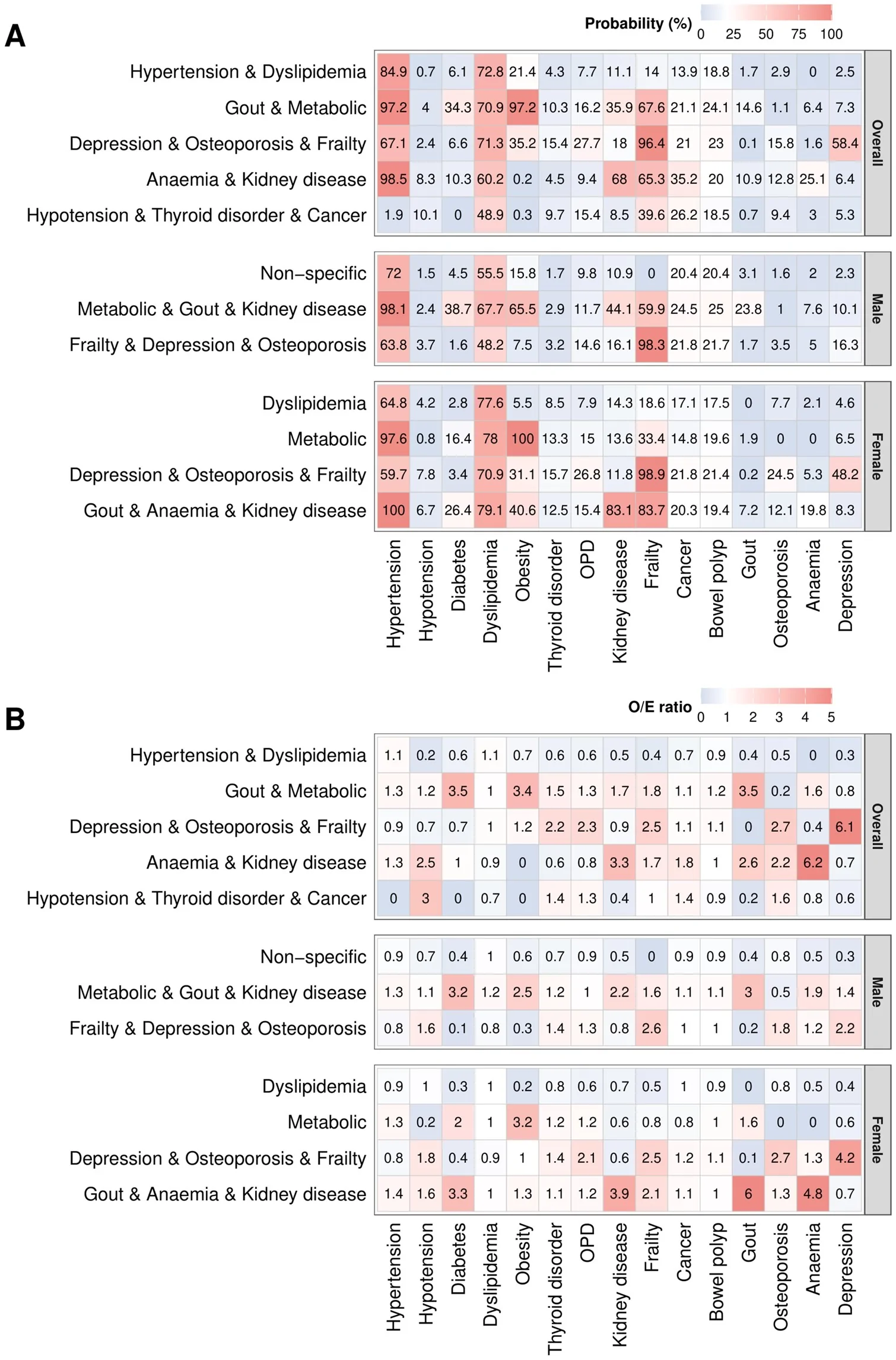
Baseline chronic condition profiles across multimorbidity clusters overall and by sex. (A) Conditional probabilities of 15 baseline chronic conditions within each cluster. (B) Observed-to-expected (O/E) ratios of chronic conditions within each cluster. “Metabolic” in cluster labels indicates obesity and diabetes. OPD, obstructive pulmonary disease.

The optimal number of clusters was 3 for males and 4 for females (Figure 1). In males, 1 cluster was labeled nonspecific because all chronic conditions had O/E ratios below 1 (*n* = 3791, 52.6%), whereas in females, the comparable low-specificity cluster (*n* = 4973, 56.0%) was mainly characterized by dyslipidemia. Depressive symptoms, osteoporosis, and frailty clustered together in both sexes, but the female cluster showed a more pronounced profile, particularly for depressive symptoms (O/E ratio 4.2 vs 2.2), as well as higher over-representation of hypotension and thyroid disorder. The remaining male cluster had over-representation of diabetes (3.2), gout (3.1), obesity (2.5), and kidney disease (2.2), whereas females had 2 additional clusters: a metabolic cluster, mainly characterized by obesity (3.2) and to a lesser extent diabetes (2.0), and a cluster with high O/E ratios for gout (6.0), anemia (4.8), and kidney disease (3.9), as well as diabetes (3.3).

Conditional probabilities of individual conditions within each cluster are shown in Figure 1 and indicate that hypertension and dyslipidemia were common across several clusters, whereas conditions such as depressive symptoms, osteoporosis, gout, anemia, kidney disease, and frailty showed more cluster-specific patterns.

### Baseline characteristics across multimorbidity clusters

Baseline characteristics varied significantly across multimorbidity clusters in the overall and sex-stratified analyses (Tables S2-S4). Participants in the gout and metabolic cluster had the highest mean disease count and were more likely to have less than 12 years of education. In sex-stratified analyses, the frailty, depressive symptoms, and osteoporosis cluster in males had the oldest age profile, while in females, it was the gout, anemia, and kidney disease cluster.

### Multimorbidity clusters and all-cause mortality

Over a median follow-up of 10.85 years (interquartile range: 9.63-12.08), 4217 deaths occurred, including 2160 in males and 2057 in females, corresponding to mortality rates of 29.35 and 22.01 per 1000 person-years, respectively (Table S6). Across the overall clusters, incidence rates ranged from 19.31 in the hypertension and dyslipidemia cluster to 42.22 in the anemia and kidney disease cluster (Table S6). Kaplan–Meier survival curves showed that 2 clusters had higher survival probability than the other 3, with significant differences across clusters (log-rank test, *p* < .001) (Figure 2).

**Figure 2:**
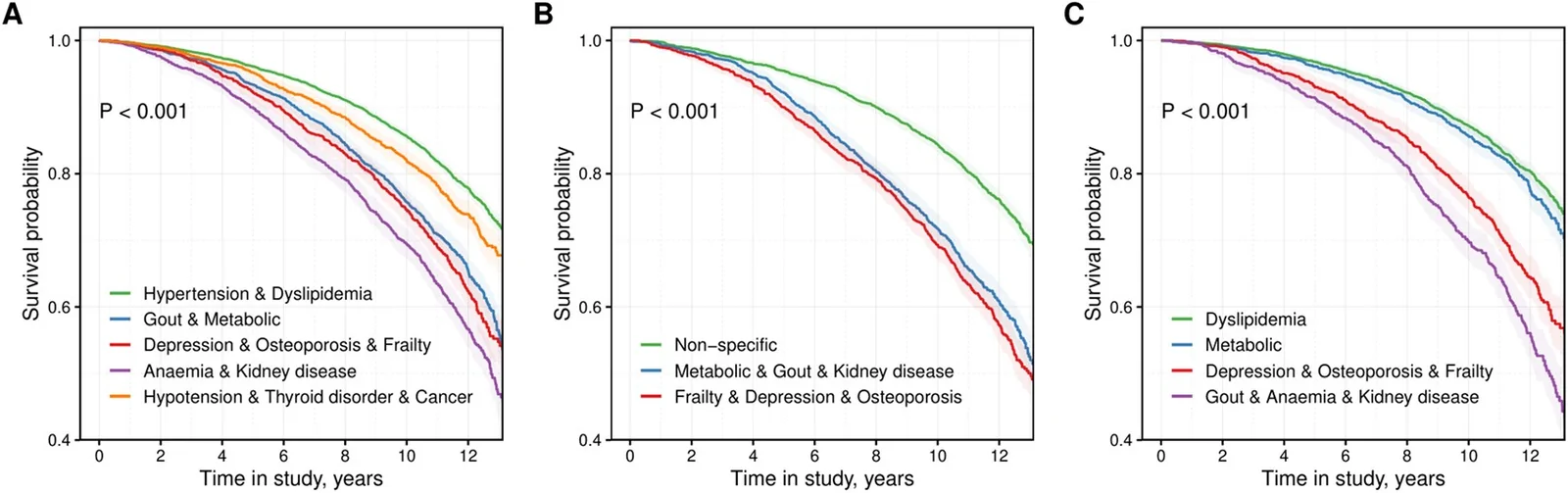
Kaplan–Meier survival curves by baseline multimorbidity cluster in the overall sample (A), males (B), and females (C). “Metabolic” in cluster labels indicates obesity and diabetes.

In the overall sample, all other clusters showed increased all-cause mortality compared with the hypertension and dyslipidemia cluster. The effect size was similar for the depressive symptoms, osteoporosis, and frailty cluster (aHR 1.68, 95% CI 1.52-1.85), the anemia and kidney disease cluster (aHR 1.65, 95% CI 1.51-1.81), and the gout and metabolic cluster (aHR 1.61, 95% CI 1.47-1.75). The hypotension, thyroid disease, and past cancer cluster showed a more modest association with mortality (aHR 1.19, 95% CI 1.08-1.31) (Figure 3). In sex-specific Cox models, relative to the nonspecific cluster in males and the dyslipidemia cluster in females, stronger mortality associations were observed for the metabolic, gout, and kidney disease cluster in males and the gout, anemia, and kidney disease cluster in females, with adjusted HRs of 1.63 (95% CI 1.47-1.81) and 1.96 (95% CI 1.74-2.21), respectively. Among females, the metabolic cluster was associated with a modestly higher mortality risk (aHR 1.20, 95% CI 1.06-1.36). The depressive symptoms, osteoporosis, and frailty cluster was also associated with higher mortality risk in both sexes, with aHRs of 1.56 (95% CI 1.40-1.73) in males and 1.68 (95% CI 1.49-1.89) in females.

**Figure 3:**
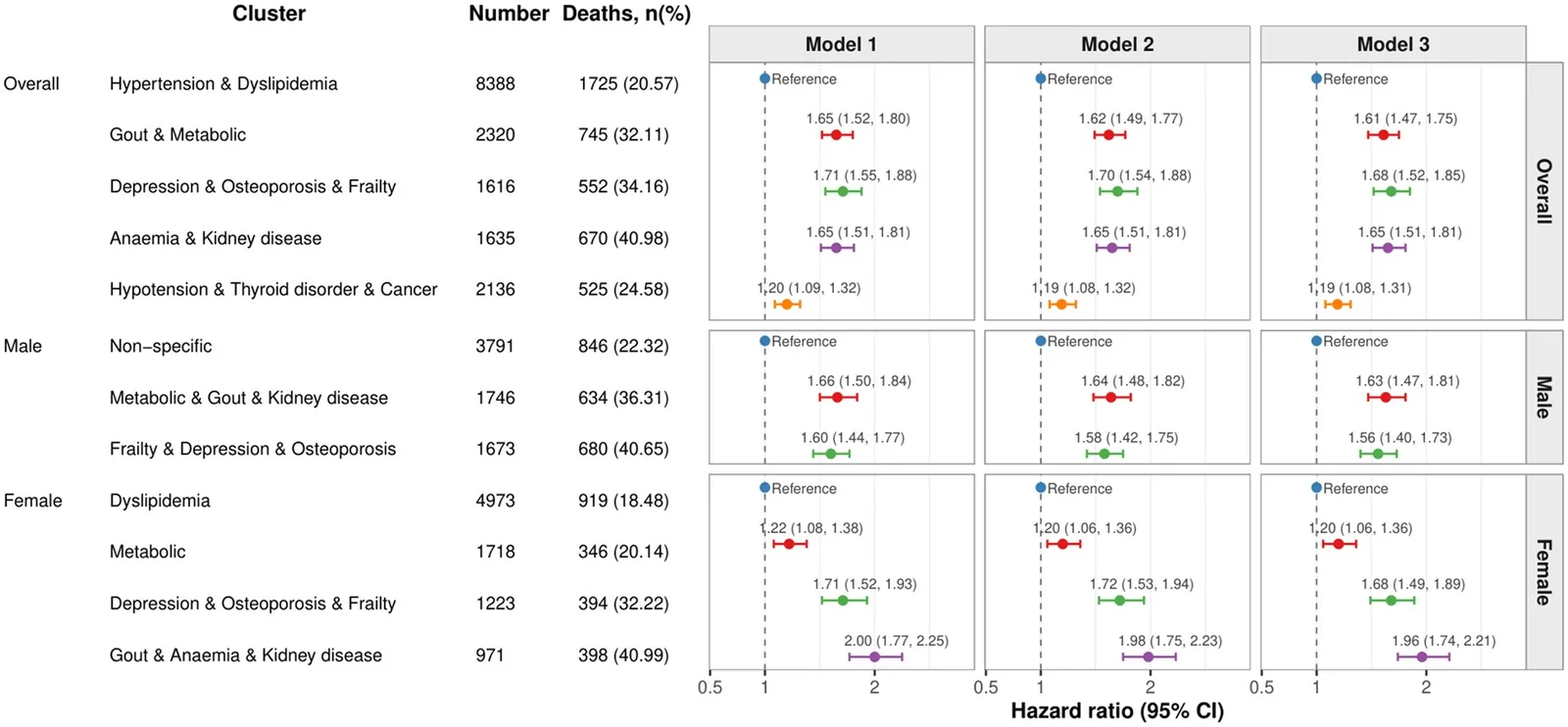
Associations between baseline multimorbidity clusters and all-cause mortality overall and by sex. “Metabolic” in cluster labels indicates obesity and diabetes. Model 1 was adjusted for age and sex in the overall sample and for age only in sex-specific analyses. Model 2 was additionally adjusted for education, residence area, and living status. Model 3 was further adjusted for smoking status and alcohol consumption.

Sensitivity analyses showed broadly similar results to the main analysis. After additional adjustment for the individual-level number of chronic conditions, associations were generally attenuated but remained consistent in the overall sample, and remained significant for all sex-specific clusters except the metabolic cluster in females (Table S7). Analyses using 5-year all-cause mortality showed similar trends in both the overall sample and by sex, with wider confidence intervals due to the smaller number of events (Figure S1).

## Discussion

In this large cohort of relatively healthy community-dwelling older adults aged 70 years and over, we applied LCA to identify probabilistic patterns of co-occurring baseline health conditions and examined their associations with mortality.^8^^,^^21^ We found that multimorbidity was highly prevalent, and the composition and mortality associations of the identified patterns varied descriptively between males and females. These sex-stratified patterns may provide prognostic information beyond the individual-level number of chronic conditions.

A key difference between our study and many previous multimorbidity studies is that the ASPREE participants were free of major cardiovascular disease at enrollment, including stroke, coronary artery disease, heart failure, and peripheral artery disease.^17^ Although cardiovascular disease remains a major cause of mortality in later life, declining age-standardized cardiovascular mortality means that an increasing number of people are reaching older age without established clinical cardiovascular disease. Previous studies have identified cardiovascular-dominant multimorbidity clusters and their strong associations with mortality.^7–9^ When established cardiovascular diseases are included in clustering analyses, they may strongly influence cluster formation and limit the characterization of less prominent clusters involving other health conditions.^22^^,^^23^ The ASPREE eligibility criteria therefore provided an opportunity to characterize multimorbidity clusters and their associations with mortality among relatively healthy older adults without established major cardiovascular disease.

The dominant clusters identified in the overall population were broadly consistent with previously reported multimorbidity clusters, including metabolic, mental health, and frailty- or physical function-related clusters.^6^^,^^8^^,^^21^^,^^22^^,^^24^^,^^25^ In the overall analysis, 5 clusters were identified, which differed in the relative representation of the included conditions. Cluster labels were used as concise summaries of relatively prominent or over-represented conditions and were not intended as exhaustive descriptions or discrete clinical phenotypes. The sex-stratified analyses further characterized the multimorbidity patterns identified separately within males and females. Consistent with previous studies, we observed clusters characterized by hypertension and dyslipidemia and by metabolic conditions, including diabetes and obesity.^8^^,^^23^ A cluster characterized by depressive symptoms, osteoporosis, and frailty was identified in the overall population and in both sex-stratified analyses, although the relative representation of its component conditions differed between males and females. Gout and kidney disease were also grouped within clusters identified separately among males and females. Among males, these conditions occurred within a broader metabolic cluster that included diabetes and obesity, whereas among females, they clustered with anemia and diabetes. These findings may partly reflect sex-related variation in the prevalence and co-occurrence of metabolic, renal, and hematological conditions.^26^

Distinct multimorbidity clusters exhibited different associations with all-cause mortality in the overall and sex-stratified analyses. In the overall population, 3 clusters showed similarly strong associations with higher mortality compared with the least distinctive reference cluster, whereas the hypotension, thyroid disorder, and cancer history cluster showed a more modest association. This may partly reflect the relatively stable or well-controlled nature of these conditions in this relatively healthy older population, as well as the presence of cancer history and thyroid disorder across multiple clusters, which may have attenuated the mortality contrast between clusters.^27^^,^^28^ Sex-stratified analyses provided further insight into cluster-specific mortality associations. In males, both non-reference clusters were associated with higher mortality, with a numerically stronger association for the metabolic, gout, and kidney disease cluster. In females, the metabolic cluster showed a more modest association, potentially reflecting the complex relationship between BMI and mortality in older adults, including reverse causation, illness-related weight loss, selective survival, and differences in body composition.^29^ The depressive symptoms, osteoporosis, and frailty cluster was associated with higher mortality in both the male and female analyses.^25^ Its composition and association with mortality may be shaped by age, hormonal changes, medication use, and the combined psychological, skeletal, and physical vulnerability represented by these conditions.^30–33^ Overall, these findings highlight the value of sex-stratified analyses for characterizing cluster–outcome associations separately within males and females, which may be less apparent in pooled analyses.^7^ The observed associations may be shaped by biological, behavioral, healthcare-related, and survival-related factors.^26^^,^^34–36^

The clusters identified by LCA represent statistical groupings of co-occurring conditions rather than distinct biological entities. These groupings may be interpreted using a framework similar to that applied to metabolic syndrome, in which related abnormalities co-occur and jointly contribute to cardiovascular risk.^37^^,^^38^ From this perspective, the observed clusters may provide clues to potential interrelationships among conditions and cumulative physiological dysregulation. The clustering of kidney disease with anemia may reflect their established clinical relationship, as impaired kidney function can contribute to anemia through reduced erythropoietin production, inflammation, and disturbances in iron metabolism.^8^^,^^39–42^ The depressive symptoms, osteoporosis, and frailty cluster may represent the convergence of mental health burden and physical vulnerability. From a clinical practice perspective, frailty present at baseline warrants assessment and management as a clinically relevant condition that can influence treatment decisions and care needs. When combined with depression and other somatic conditions, frailty may contribute to poorer outcomes, including mortality.^31^ The cluster characterized by hypotension, thyroid disorder, and cancer history has been less commonly reported in previous studies. Although hypotension is less frequently included in clustering studies, among older adults it may reflect impaired hemodynamic regulation, medication effects, or underlying disease burden, while thyroid disorder and cancer history may add further clinical complexity.^43–45^ Taken together, this cluster may represent a heterogeneous grouping of health conditions rather than a distinct clinical entity. Further research is needed to validate the reproducibility and prospective predictive value of these clusters before they can be considered for clinical screening or intervention.

Beyond their associations with mortality, multimorbidity clusters may have implications for research and, with further validation, clinical care. Identifying frequently co-occurring conditions can generate hypotheses about shared disease mechanisms, common risk pathways, and the natural history of multimorbidity.^23^ Clinically, validated cluster information could help identify coexisting conditions that might otherwise be overlooked and support more person-centered assessment of treatment goals, medication burden, functional status, and patient priorities.^10^^,^^21^^,^^36^ If shown to be reproducible and prospectively predictive, these clusters may eventually inform screening approaches, intervention strategies, clinical guidelines, and trial designs that better reflect the complexity of multimorbidity in older adults.^36^ The clusters identified separately within males and females also suggest that future validation should consider sex-stratified analyses.

This study has several strengths. The large, community-dwelling ASPREE cohort and long follow-up enabled robust examination of multimorbidity clusters and their associations with mortality among adults aged 70 years and over. The ASPREE eligibility criteria also enabled examination of these associations among relatively healthy individuals who had reached later life without established major cardiovascular disease. The use of LCA supported the data-driven identification of probabilistic clusters, while analyses conducted separately within males and females characterized the clusters and their mortality associations within each sex.

Several limitations should be noted. First, the selected ASPREE cohort comprised relatively healthy older adults, limiting generalizability to those with established cardiovascular disease, dementia, major physical disability, or greater clinical burden. Second, only 15 indicators were included, and alternative indicators may have resulted in a different number or composition of clusters. Third, disease severity was not captured, and class assignment showed some uncertainty and overlap; the clusters should therefore be interpreted as probabilistic groupings rather than discrete clinical phenotypes. Fourth, clusters were based only on baseline conditions and did not capture incident conditions, disease progression, treatment changes, or transitions between clusters over time. Finally, residual confounding from unmeasured factors may have influenced the observed associations.

In conclusion, distinct multimorbidity clusters were identified among relatively healthy older adults, both overall and separately in males and females. Several clusters, including those characterized by frailty, depressive symptoms, osteoporosis, or kidney disease, were associated with higher all-cause mortality. These findings are hypothesis-generating and support further investigation of the relationship between multimorbidity clustering and mortality in older adults.

## Supplementary Material

glag245_Supplementary_Data

## Data Availability

The data used in this study are not publicly available owing to participant privacy and ethical restrictions. Information regarding requests for access to ASPREE data is available through the ASPREE website.

## References

[1] Ho ISS , Azcoaga-Lorenzo A, Akbari A, et al Measuring multimorbidity in research: Delphi consensus study. BMJMED. 2022;1:e000247. 10.1136/bmjmed-2022-000247 PMC9978673 36936594

[2] Ho ISS , Azcoaga-Lorenzo A, Akbari A, et al Variation in the estimated prevalence of multimorbidity: systematic review and meta-analysis of 193 international studies. BMJ Open. 2022;12:e057017. 10.1136/bmjopen-2021-057017 PMC9058768 35487738

[3] Prados-Torres A , Calderón-Larrañaga A, Hancco-Saavedra J, Poblador-Plou B, van den Akker M. Multimorbidity patterns: a systematic review. J Clin Epidemiol. 2014;67:254-266. 10.1016/j.jclinepi.2013.09.021 24472295

[4] Fried TR , Tinetti ME, Iannone L, O’Leary JR, Towle V, Van Ness PH. Health outcome prioritization as a tool for decision making among older persons with multiple chronic conditions. Arch Intern Med. 2011;171:1854-1856. 10.1001/archinternmed.2011.424 PMC4036681 21949032

[5] Johnston MC , Crilly M, Black C, Prescott GJ, Mercer SW. Defining and measuring multimorbidity: a systematic review of systematic reviews. Eur J Public Health. 2019;29:182-189. 10.1093/eurpub/cky098 29878097

[6] Xi J , Miao M, Li PWC, Yu DSF. Prognostic effects of multimorbidity clusters on health outcomes in adults: a systematic review and meta-analysis. Ageing Res Rev. 2025;112:102897. 10.1016/j.arr.2025.102897 40934974

[7] Ferrer A , Formiga F, Sanz H, Almeda J, Padrós G. Multimorbidity as specific disease combinations, an important predictor factor for mortality in octogenarians: the Octabaix study. Clin Interv Aging. 2017;12:223-231. 10.2147/CIA.S123173 PMC5291453 28184153

[8] Ferris JK , Fiedeldey LK, Kim B, et al A systematic review and meta-analysis of disease clusters in multimorbidity. Nat Commun. 2025;17:634. 10.1038/s41467-025-67372-6 PMC12816623 41453855

[9] Nichols L , Taverner T, Crowe F, et al In simulated data and health records, latent class analysis was the optimum multimorbidity clustering algorithm. J Clin Epidemiol. 2022;152:164-175. 10.1016/j.jclinepi.2022.10.011 PMC7613854 36228971

[10] Tran T , Bliuc D, Ho-Le T, et al Association of multimorbidity and excess mortality after fractures among Danish adults. JAMA Netw Open. 2022;5:e2235856. 10.1001/jamanetworkopen.2022.35856 PMC9552889 36215068

[11] Nylund KL , Asparouhov T, Muthén BO. Deciding on the number of classes in latent class analysis and growth mixture modeling: a Monte Carlo simulation study. Struct Equat Model. 2007;14:535-569. 10.1080/10705510701575396

[12] Makovski TT , Schmitz S, Zeegers MP, Stranges S, van den Akker M. Multimorbidity and quality of life: systematic literature review and meta-analysis. Ageing Res Rev. 2019;53:100903. 10.1016/j.arr.2019.04.005 31048032

[13] Chowdhury SR , Das DC, Sunna TC, Beyene J, Hossain A. Global and regional prevalence of multimorbidity in the adult population in community settings: a systematic review and meta-analysis. EClinicalMedicine. 2023;57:101860. 10.1016/j.eclinm.2023.101860 PMC9971315 36864977

[14] Kabir A , Conway DP, Ansari S, Tran A, Rhee JJ, Barr M. Impact of multimorbidity and complex multimorbidity on healthcare utilisation in older Australian adults aged 45 years or more: a large population-based cross-sectional data linkage study. *BMJ Open*. 2024;14(1):e078762. 10.1136/bmjopen-2023-078762 PMC10806611 38199624

[15] McNeil JJ , Woods RL, Nelson MR, et al Baseline characteristics of participants in the ASPREE (ASPirin in Reducing Events in the Elderly) study. J Gerontol A Biol Sci Med Sci. 2017;72:1586-1593. 10.1093/gerona/glw342 PMC5861878 28329340

[16] Ernst ME , Broder JC, Wolfe R, et al Health characteristics and aspirin use in participants at the baseline of the ASPirin in Reducing Events in the Elderly–eXTension (ASPREE-XT) observational study. Contemp Clin Trials. 2023;130:107231. 10.1016/j.cct.2023.107231 PMC10330669 37196887

[17] McNeil JJ , Woods RL, Nelson MR, et al Effect of aspirin on disability-free survival in the healthy elderly. N Engl J Med. 2018;379:1499-1508. 10.1056/NEJMoa1800722 PMC6426126 30221596

[18] Stocks NP , González-Chica DA, Woods RL, et al Quality of life for 19,114 participants in the ASPREE (ASPirin in Reducing Events in the Elderly) study and their association with sociodemographic and modifiable lifestyle risk factors. Qual Life Res. 2019;28:935-946. 10.1007/s11136-018-2040-z PMC6924574 30411180

[19] McNeil JJ , Nelson MR, Woods RL, et al Effect of aspirin on all-cause mortality in the healthy elderly. N Engl J Med. 2018;379:1519-1528. 10.1056/NEJMoa1803955 PMC6433466 30221595

[20] Goodman LA. Exploratory latent structure analysis using both identifiable and unidentifiable models. Biometrika. 1974;61:215-231. 10.1093/biomet/61.2.215

[21] Busija L , Lim K, Szoeke C, Sanders KM, McCabe MP. Do replicable profiles of multimorbidity exist? Systematic review and synthesis. Eur J Epidemiol. 2019;34:1025-1053. 10.1007/s10654-019-00568-5 31624969

[22] He K , Zhang W, Hu X, et al Relationship between multimorbidity, disease cluster and all-cause mortality among older adults: a retrospective cohort analysis. BMC Public Health. 2021;21:1080. 10.1186/s12889-021-11108-w PMC8180153 34090390

[23] Alvarez-Galvez J , Ortega-Martin E, Ramos-Fiol B, Suarez-Lledo V, Carretero-Bravo J. Epidemiology, mortality, and health service use of local-level multimorbidity patterns in South Spain. Nat Commun. 2023;14:7689. 10.1038/s41467-023-43569-5 PMC10673852 38001107

[24] Moussavi S , Chatterji S, Verdes E, Tandon A, Patel V, Ustun B. Depression, chronic diseases, and decrements in health: results from the World Health Surveys. Lancet. 2007;370:851-858. 10.1016/S0140-6736(07)61415-9 17826170

[25] DeLong LN , Fleetwood K, Prigge R, Galdi P, Guthrie B, Fleuriot JD. Cluster and survival analysis of UK biobank data reveals associations between physical multimorbidity clusters and subsequent depression. Commun Med (Lond). 2025;5:156. 10.1038/s43856-025-00825-7 PMC12075648 40360733

[26] Arshadipour A , Thorand B, Linkohr B, Ladwig KH, Heier M, Peters A. Multimorbidity patterns and mortality in older adults: results from the KORA-Age study. Front Nutr. 2023;10:1146442. 10.3389/fnut.2023.1146442 PMC10083328 37051131

[27] Ogliari G , Smit RAJ, van der Spoel E, et al Thyroid status and mortality risk in older adults with normal thyrotropin: sex differences in the Milan geriatrics 75+ cohort study. J Gerontol A Biol Sci Med Sci. 2017;72:554-559. 10.1093/gerona/glw113 27371954

[28] Koczwara B , Meng R, Miller MD, et al Late mortality in people with cancer: a population‐based Australian study. Med J Aust. 2021;214. https://www.mja.com.au/journal/2021/214/7/late-mortality-people-cancer-population-based-australian-study? utm_source=chatgpt.com 10.5694/mja2.50879 33296507

[29] Dramé M , Godaert L. The obesity paradox and mortality in older adults: a systematic review. Nutrients. 2023;15:1780. 10.3390/nu15071780 PMC10096985 37049633

[30] Bobo WV , Grossardt BR, Virani S, St Sauver JL, Boyd CM, Rocca WA. Association of depression and anxiety with the accumulation of chronic conditions. JAMA Netw Open. 2022;5:e229817. 10.1001/jamanetworkopen.2022.9817 PMC9062691 35499825

[31] Velek P , Luik AI, Brusselle GGO, et al Sex-specific patterns and lifetime risk of multimorbidity in the general population: a 23-year prospective cohort study. BMC Med. 2022;20:304. 10.1186/s12916-022-02487-x PMC9454172 36071423

[32] Bobo WV , Yawn BP, St Sauver JL, Grossardt BR, Boyd CM, Rocca WA. Prevalence of combined somatic and mental health multimorbidity: patterns by age, sex, and race/ethnicity. J Gerontol A Biol Sci Med Sci. 2016;71:1483-1491. 10.1093/gerona/glw032 PMC5055644 26935110

[33] Dumbreck S , Flynn A, Nairn M, et al Drug-disease and drug-drug interactions: systematic examination of recommendations in 12 UK national clinical guidelines. BMJ. 2015;350:h949. 10.1136/bmj.h949 PMC4356453 25762567

[34] Roman Lay AA , Ferreira do Nascimento C, Caba Burgos F, et al Gender differences between multimorbidity and all-cause mortality among older adults. Curr Gerontol Geriatr Res. 2020;2020:7816785. 10.1155/2020/7816785 PMC7049854 32148480

[35] Kc R , Voigt R, Li X, et al Induction of osteoarthritis-like pathologic changes by chronic alcohol consumption in an experimental mouse model. Arthritis Rheumatol. 2015;67:1678-1680. 10.1002/art.39090 PMC4823794 25708245

[36] Kuan V , Denaxas S, Patalay P, et al Identifying and visualising multimorbidity and comorbidity patterns in patients in the English National Health Service: a population-based study. Lancet Digit Health. 2023;5:e16-e27. 10.1016/S2589-7500(22)00187-X 36460578

[37] Alberti KGMM , Eckel RH, Grundy SM, et al Harmonizing the metabolic syndrome: a joint interim statement of the International Diabetes Federation Task Force on Epidemiology and Prevention; National Heart, Lung, and Blood Institute; American Heart Association; World Heart Federation; International Atherosclerosis Society; and International Association for the Study of Obesity. Circulation. 2009;120:1640-1645. 10.1161/CIRCULATIONAHA.109.192644 19805654

[38] Alberti KGMM , Zimmet P, Shaw J. Metabolic syndrome—a new world-wide definition. A consensus statement from the International Diabetes Federation. Diabet Med. 2006;23:469-480. 10.1111/j.1464-5491.2006.01858.x 16681555

[39] Agarwal AK. Iron metabolism and management: focus on chronic kidney disease. Kidney Int Suppl (2011). 2021;11:46-58. 10.1016/j.kisu.2020.12.003 PMC7983022 33777495

[40] Badura K , Janc J, Wąsik J, et al Anemia of chronic kidney disease—a narrative review of its pathophysiology, diagnosis, and management. Biomedicines. 2024;12:1191. 10.3390/biomedicines12061191 PMC11200696 38927397

[41] Tarng DC. Cardiorenal anemia syndrome in chronic kidney disease. J Chin Med Assoc. 2007;70:424-429. 10.1016/S1726-4901(08)70031-5 17962140

[42] Corsonello A , Fabbietti P, Formiga F, et al Chronic kidney disease in the context of multimorbidity patterns: the role of physical performance: the screening for CKD among older people across Europe (SCOPE) study. BMC Geriatr. 2020;20:350. 10.1186/s12877-020-01696-4 PMC7532089 33008303

[43] Morrissey Y , Bedford M, Irving J, Farmer CKT. Older people remain on blood pressure agents despite being hypotensive resulting in increased mortality and hospital admission. Age Ageing. 2016;45:783-788. 10.1093/ageing/afw120 27496937

[44] Lee Y. Orthostatic hypotension in older people. J Am Assoc Nurse Pract. 2013;25:451-458. 10.1002/2327-6924.12026 24170648

[45] Farrell MC , Shibao CA. Morbidity and mortality in orthostatic hypotension. Auton Neurosci. 2020;229:102717. 10.1016/j.autneu.2020.102717 32896712

