# Supplementary material for: Sex-specific multimorbidity clusters and all-cause mortality among community-dwelling older adults: findings from the ASPREE cohort
Source: J Gerontol A Biol Sci Med Sci. 2026 Oct 1;81(11):glag245. doi: 10.1093/gerona/glag245 (PMC13680096; doi:10.1093/gerona/glag245)
Supplement: Supplementary file 1 [file glag245_supplementary_data.pdf]

Sex-specific multimorbidity clusters and all-cause mortality among community-dwelling older adults: findings from the ASPREE cohort

Supplementary tables and figures 1

eTable 1. Definitions of the 15 baseline chronic conditions included in the analysis 1

eTable 2. Baseline characteristics of participants according to multimorbidity clusters in the overall sample 2

eTable 3. Baseline characteristics of male participants according to multimorbidity clusters 4

eTable 4. Baseline characteristics of female participants according to multimorbidity clusters 5

eTable 5. Model fit and classification diagnostics for candidate latent class solutions in the overall and sex-stratified samples 6

eTable 6. Mortality incidence rates across baseline multimorbidity clusters during follow-up 7

eTable 7. Sensitivity analysis of the association between baseline multimorbidity clusters and all-cause mortality after additional adjustment for the individual-level number of chronic conditions 8

eFigure 1. Associations between baseline multimorbidity clusters and 5-year all-cause mortality in the overall sample and by sex. 9

Supplementary tables and figures

eTable 1. Definitions of the 15 baseline chronic conditions included in the analysis

Condition	Definition
Hypertension	Mean of three seated blood pressure measurements with a mean systolic blood pressure (SBP) ≥ 140 mmHg, or mean diastolic blood pressure (DBP) ≥ 90 mmHg, or current use of antihypertensive medication.
Hypotension	Mean of three seated blood pressure measurements with a mean SBP < 90 mmHg or mean DBP < 60 mmHg.
Diabetes	Self-reported diabetes, fasting blood glucose > 125 mg/dL, or current pharmaceutical treatment for diabetes.
Dyslipidemia	Use of cholesterol-lowering medication, serum total cholesterol ≥ 212 mg/dL, or low-density lipoprotein cholesterol > 160 mg/dL (> 4.1 mmol/L).
Obesity	Body mass index (BMI) ≥ 30 kg/m^2.
Thyroid disorder	Current use of medication for thyroid disorder.
Obstructive pulmonary disease	Self-reported asthma or current use of medication for obstructive pulmonary disease.
Kidney disease	Self-reported kidney disease, estimated glomerular filtration rate (eGFR) <60 mL/min/1.73 m^2, or spot urine albumin-to-creatinine ratio (ACR) ≥3 mg/mmol.
Pre-Frailty/Frailty	Defined as a Fried frailty phenotype score ≥1, including both pre-frail and frail participants.
Cancer history	Self-reported history of cancer.
Bowel polyp	Self-reported history of bowel polyp.
Gout	Self-reported gout or current use of medication for gout.
Osteoporosis	Use of osteoporosis-related medication.
Anaemia	Haemoglobin < 13 g/dL in men and < 12 g/dL in women.
Depressive symptoms	Center for Epidemiologic Studies Depression Scale 10-item version (CESD-10) score ≥ 8.

eTable 2. Baseline characteristics of participants according to multimorbidity clusters in the overall sample

**Characteristics**	**Hypertension & Dyslipidemia N = 8,388**	**Gout & Metabolic N = 2,320**	**Depression, Osteoporosis & Frailty N = 1,616**	**Anaemia & Kidney disease N = 1,635**	**Hypotension, Thyroid disorder & Cancer N = 2,136**	**P value**
Age, mean (SD)	74.71 (3.94)	75.34 (4.22)	76.08 (4.56)	77.89 (5.30)	74.97 (4.30)	<0.001
Sex, n (%)						<0.001
Female	4523 (53.92)	1257 (54.18)	1117 (69.12)	835 (51.07)	1153 (53.98)	
Male	3865 (46.08)	1063 (45.82)	499 (30.88)	800 (48.93)	983 (46.02)	
Age group, n (%)						<0.001
70–74	5301 (63.20)	1294 (55.78)	802 (49.63)	600 (36.70)	1321 (61.84)	
75–79	2104 (25.08)	678 (29.22)	490 (30.32)	485 (29.66)	517 (24.20)	
80+	983 (11.72)	348 (15.00)	324 (20.05)	550 (33.64)	298 (13.95)	
Education, n (%)						<0.001
≥12 years	4344 (51.79)	969 (41.77)	764 (47.28)	787 (48.13)	1142 (53.46)	
<12 years	4044 (48.21)	1351 (58.23)	852 (52.72)	848 (51.87)	994 (46.54)	
Residence area, n (%)						<0.001
Outer region	1026 (12.23)	308 (13.28)	138 (8.54)	172 (10.52)	242 (11.33)	
Inner region	3064 (36.53)	844 (36.38)	572 (35.40)	550 (33.64)	713 (33.38)	
Major cities	4298 (51.24)	1168 (50.34)	906 (56.06)	913 (55.84)	1181 (55.29)	
Living status, n (%)						<0.001
Living with someone	5980 (71.29)	1525 (65.73)	970 (60.02)	1031 (63.06)	1463 (68.49)	
Living alone at home	2408 (28.71)	795 (34.27)	646 (39.98)	604 (36.94)	673 (31.51)	
Smoking status, n (%)						<0.001
Current	275 (3.28)	68 (2.93)	66 (4.08)	45 (2.75)	90 (4.21)	
Former	3387 (40.38)	1047 (45.13)	679 (42.02)	697 (42.63)	772 (36.14)	
Never	4726 (56.34)	1205 (51.94)	871 (53.90)	893 (54.62)	1274 (59.64)	
Alcohol consumption, n (%)						<0.001
Current	6775 (80.77)	1692 (72.93)	1238 (76.61)	1270 (77.68)	1725 (80.76)	
Former	345 (4.11)	151 (6.51)	91 (5.63)	100 (6.12)	94 (4.40)	
Never	1268 (15.12)	477 (20.56)	287 (17.76)	265 (16.21)	317 (14.84)	
Chronic condition count, n (%)						<0.001

0	0 (0.00)	0 (0.00)	0 (0.00)	0 (0.00)	319 (14.93)	
1	1197 (14.27)	0 (0.00)	0 (0.00)	0 (0.00)	374 (17.51)	
≥2	7191 (85.73)	2320 (100.00)	1616 (100.00)	1635 (100.00)	1443 (67.56)	
Chronic condition count, mean (SD)	2.63 (1.02)	5.08 (1.25)	4.60 (1.29)	4.35 (1.07)	1.98 (1.24)	<0.001

Note: “Metabolic” in the labels indicates obesity and diabetes.

eTable 3. Baseline characteristics of male participants according to multimorbidity clusters

Characteristics	Non-specific (N = 3,791)	Metabolic, Gout & Kidney disease (N = 1,746)	Frailty, Depression & Osteoporosis (N = 1,673)	P value
Age, mean (SD)	74.29 (3.66)	75.49 (4.44)	76.64 (5.05)	<0.001
Age group, n (%)				<0.001
70–74	2564 (67.63)	960 (54.98)	790 (47.22)	
75–79	865 (22.82)	504 (28.87)	463 (27.67)	
80+	362 (9.55)	282 (16.15)	420 (25.10)	
Education, n (%)				<0.001
≥12 years	2123 (56.00)	824 (47.19)	871 (52.06)	
<12 years	1668 (44.00)	922 (52.81)	802 (47.94)	
Residence area, n (%)				0.133
Outer region	434 (11.45)	206 (11.80)	191 (11.42)	
Inner region	1408 (37.14)	617 (35.34)	563 (33.65)	
Major cities	1949 (51.41)	923 (52.86)	919 (54.93)	
Living status, n (%)				<0.001
Living with someone	3152 (83.14)	1359 (77.84)	1250 (74.72)	
Living alone at home	639 (16.86)	387 (22.16)	423 (25.28)	
Smoking status, n (%)				<0.001
Current	134 (3.53)	61 (3.49)	83 (4.96)	
Former	1914 (50.49)	1023 (58.59)	898 (53.68)	
Never	1743 (45.98)	662 (37.92)	692 (41.36)	
Alcohol consumption, n (%)				0.001
Current	3294 (86.89)	1469 (84.14)	1392 (83.20)	
Former	187 (4.93)	123 (7.04)	111 (6.63)	
Never	310 (8.18)	154 (8.82)	170 (10.16)	
Chronic condition count, n (%)				<0.001
0	204 (5.38)	0 (0.00)	0 (0.00)	
1	759 (20.02)	0 (0.00)	72 (4.30)	
≥2	2828 (74.60)	1746 (100.00)	1601 (95.70)	
Chronic condition count, mean (SD)	2.22 (1.09)	4.83 (1.24)	3.27 (1.20)	<0.001

Note: "Metabolic" in the labels indicates obesity and diabetes.

eTable 4. Baseline characteristics of female participants according to multimorbidity clusters

Characteristics	Dyslipidemia (N = 4,973)	Metabolic (N = 1,718)	Depression, Osteoporosis & Frailty (N = 1,223)	Gout, Anaemia & Kidney disease (N = 971)	P value
Age, mean (SD)	75.05 (4.14)	74.56 (3.68)	76.28 (4.67)	77.90 (5.28)	<0.001
Age group, n (%)					<0.001
70–74	2971 (59.74)	1097 (63.85)	590 (48.24)	346 (35.63)	
75–79	1311 (26.36)	456 (26.54)	370 (30.25)	305 (31.41)	
80+	691 (13.90)	165 (9.60)	263 (21.50)	320 (32.96)	
Education, n (%)					<0.001
≥12 years	2480 (49.87)	748 (43.54)	571 (46.69)	389 (40.06)	
<12 years	2493 (50.13)	970 (56.46)	652 (53.31)	582 (59.94)	
Residence area, n (%)					0.005
Outer region	599 (12.05)	227 (13.21)	111 (9.08)	118 (12.15)	
Inner region	1748 (35.15)	635 (36.96)	421 (34.42)	351 (36.15)	
Major cities	2626 (52.81)	856 (49.83)	691 (56.50)	502 (51.70)	
Living status, n (%)					<0.001
Living with someone	3021 (60.75)	999 (58.15)	674 (55.11)	514 (52.94)	
Living alone at home	1952 (39.25)	719 (41.85)	549 (44.89)	457 (47.06)	
Smoking status, n (%)					<0.001
Current	156 (3.14)	32 (1.86)	57 (4.66)	21 (2.16)	
Former	1491 (29.98)	558 (32.48)	407 (33.28)	291 (29.97)	
Never	3326 (66.88)	1128 (65.66)	759 (62.06)	659 (67.87)	
Alcohol consumption, n (%)					<0.001
Current	3836 (77.14)	1178 (68.57)	900 (73.59)	631 (64.98)	
Former	169 (3.40)	82 (4.77)	63 (5.15)	46 (4.74)	
Never	968 (19.47)	458 (26.66)	260 (21.26)	294 (30.28)	
Chronic condition count, n (%)					<0.001
0	115 (2.31)	0 (0.00)	0 (0.00)	0 (0.00)	
1	740 (14.88)	0 (0.00)	0 (0.00)	0 (0.00)	
≥2	4118 (82.81)	1718 (100.00)	1223 (100.00)	971 (100.00)	
Chronic condition count, mean (SD)	2.53 (1.11)	4.11 (1.11)	4.47 (1.23)	5.35 (1.29)	<0.001

Note: "Metabolic" in the labels indicates obesity and diabetes.

eTable 5. Model fit and classification diagnostics for candidate latent class solutions in the overall and sex-stratified samples

Sample	Classes	AIC	BIC	Log likelihood	G²	Entropy	Classification error	AvePP	Minimum assigned class, %
**Overall**	2	189,592.0	189,830.3	-94765.02	6,113.4	0.339	0.218	0.782	32.8
	3	189,059.4	189,420.6	-94482.69	5,548.7	0.405	0.285	0.715	15.8
	4	188,873.6	189,357.8	-94373.79	5,330.9	0.415	0.317	0.683	11.0
	**5***	**188,749.5**	**189,356.7**	**-94295.75**	**5,174.9**	**0.411**	**0.380**	**0.620**	**10.0**
	6	188,664.2	189,394.4	-94237.11	5,057.6	0.363	0.453	0.547	9.9
	7	188,578.6	189,431.8	-94178.31	4,940.0	0.433	0.431	0.569	3.4
	8	188,526.8	189,503.0	-94136.41	4,856.2	0.466	0.432	0.568	2.5
	9	188,500.2	189,599.3	-94107.10	4,797.6	0.501	0.428	0.572	2.0
	10	188,482.1	189,704.3	-94082.07	4,747.5	0.494	0.463	0.537	1.6
**Male**	2	82,293.1	82,506.4	-41115.53	3,365.6	0.370	0.205	0.795	31.2
	**3***	**82,123.5**	**82,447.0**	**-41014.77**	**3,164.0**	**0.403**	**0.269**	**0.731**	**23.2**
	4	82,033.5	82,467.1	-40953.73	3,042.0	0.420	0.329	0.671	5.8
	5	81,982.5	82,526.3	-40912.27	2,959.0	0.449	0.364	0.636	2.3
	6	81,949.7	82,603.6	-40879.86	2,894.2	0.502	0.375	0.625	2.2
	7	81,928.4	82,692.4	-40853.19	2,840.9	0.493	0.380	0.620	2.5
	8	81,920.7	82,794.9	-40833.35	2,801.2	0.467	0.419	0.581	2.1
**Female**	2	104,802.1	105,021.9	-52370.04	4,557.0	0.344	0.216	0.784	32.9
	3	104,481.2	104,814.5	-52193.61	4,204.1	0.424	0.271	0.729	20.3
	**4***	**104,357.8**	**104,804.6**	**-52115.88**	**4,048.7**	**0.528**	**0.269**	**0.731**	**10.9**
	5	104,277.8	104,838.1	-52059.92	3,936.8	0.434	0.384	0.616	10.0
	6	104,255.2	104,928.9	-52032.59	3,882.1	0.445	0.392	0.608	7.0
	7	104,234.3	105,021.5	-52006.13	3,829.2	0.432	0.446	0.554	5.9
	8	104,222.0	105,122.7	-51983.98	3,784.9	0.639	0.270	0.730	1.2

Note: *Selected solution. AIC, Akaike information criterion; BIC, Bayesian information criterion; G², likelihood-ratio chi-square statistic; AvePP, average posterior probability of assigned class.

eTable 6. Mortality incidence rates across baseline multimorbidity clusters during follow-up

Multimorbidity cluster	Deaths / person-years	Incidence rate per 1,000 person-years (95% CI)
**Overall**	**4,217 / 167,030**	**25.25 (24.49–26.02)**
Hypertension & Dyslipidemia	1,725 / 89,348	19.31 (18.41–20.24)
Gout & Metabolic	745 / 23,381	31.86 (29.62–34.24)
Depression, Osteoporosis & Frailty	552 / 16,111	34.26 (31.46–37.24)
Anaemia & Kidney disease	670 / 15,867	42.22 (39.09–45.55)
Hypotension, Thyroid disorder & Cancer	525 / 22,323	23.52 (21.55–25.62)
**Male**	**2,160 / 73,583**	**29.35 (28.13–30.62)**
Non-specific	846 / 40,062	21.12 (19.72–22.59)
Metabolic, Gout & Kidney disease	634 / 17,128	37.02 (34.19–40.01)
Frailty, Depression & Osteoporosis	680 / 16,393	41.48 (38.42–44.72)
**Female**	**2,057 / 93,447**	**22.01 (21.07–22.98)**
Dyslipidemia	919 / 53,372	17.22 (16.12–18.37)
Metabolic	346 / 18,199	19.01 (17.06–21.12)
Depression, Osteoporosis & Frailty	394 / 12,372	31.85 (28.78–35.15)
Gout, Anaemia & Kidney disease	398 / 9,504	41.88 (37.86–46.20)

Note: “Metabolic” in the labels indicates obesity and diabetes.

eTable 7. Sensitivity analysis of the association between baseline multimorbidity clusters and all-cause mortality after additional adjustment for the individual-level number of chronic conditions

Multimorbidity cluster	aHR (95% CI)	P value
**Overall**		
Hypertension & Dyslipidemia	1.00 (Ref.)	
Gout & Metabolic	1.34 (1.20–1.49)	<0.001
Depression, Osteoporosis & Frailty	1.46 (1.31–1.63)	<0.001
Anaemia & Kidney disease	1.46 (1.32–1.61)	<0.001
Hypotension, Thyroid disorder & Cancer	1.25 (1.13–1.38)	<0.001
**Male**		
Non-specific	1.00 (Ref.)	
Metabolic, Gout & Kidney disease	1.38 (1.20–1.59)	<0.001
Frailty & Depression & Osteoporosis	1.46 (1.31–1.63)	<0.001
**Female**		
Dyslipidemia	1.00 (Ref.)	
Metabolic	1.09 (0.95–1.25)	0.216
Depression, Osteoporosis & Frailty	1.50 (1.30–1.71)	<0.001
Gout, Anaemia & Kidney disease	1.67 (1.43–1.94)	<0.001

Note: "Metabolic" in the labels indicates obesity and diabetes. Cox proportional hazards models were adjusted for sex (overall sample) and for age, education, residence area, living status, smoking status, alcohol consumption, and number of chronic conditions.

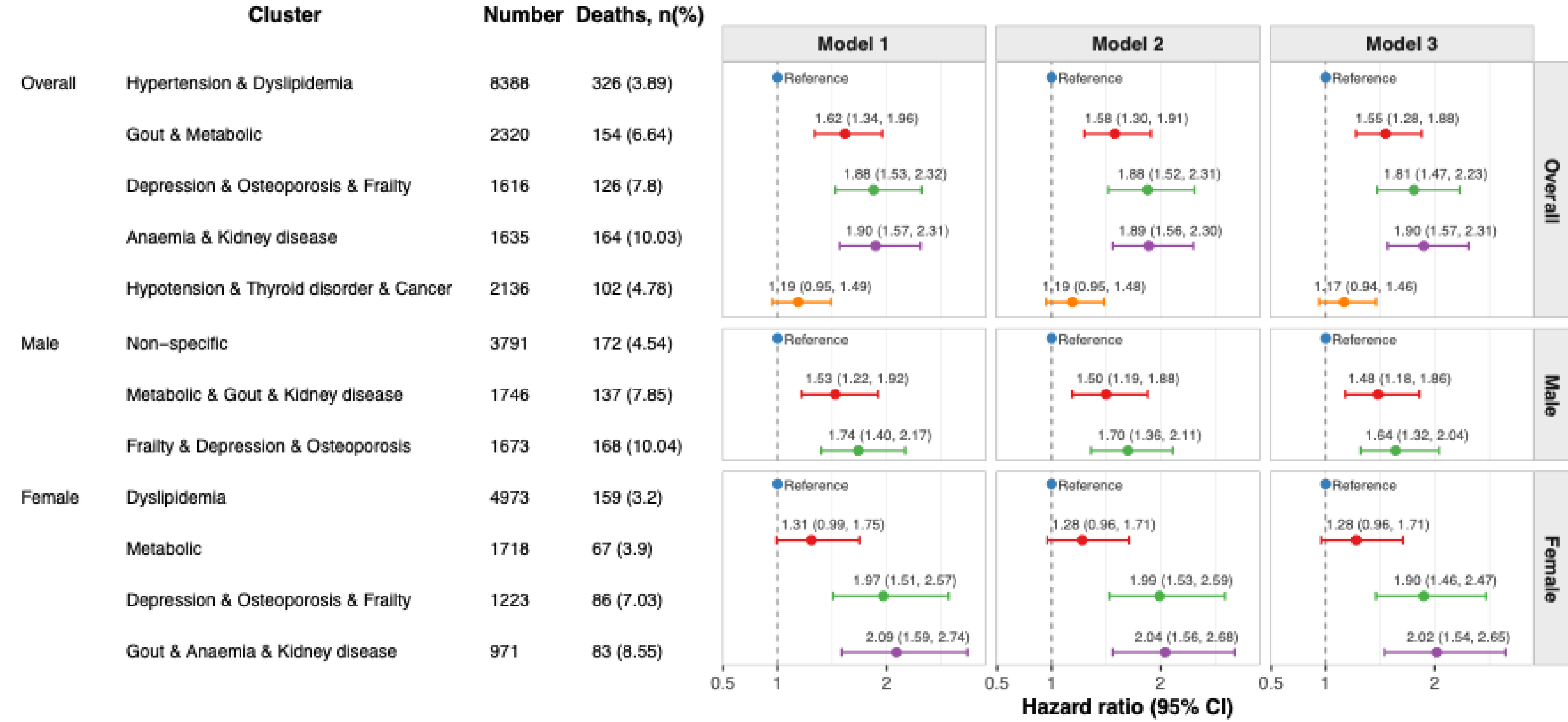

eFigure 1. Associations between baseline multimorbidity clusters and 5-year all-cause mortality in the overall sample and by sex.

Note: “Metabolic” in the labels indicates obesity and diabetes. Model 1 was adjusted for age and sex (overall sample) or age only (sex-specific analyses). Model 2 was additionally adjusted for education, residence area, and living status. Model 3 was further adjusted for smoking status and alcohol consumption.
